# Application of Electro-Fenton Technology to Remediation of Polluted Effluents by Self-Sustaining Process

**DOI:** 10.1155/2014/801870

**Published:** 2014-02-26

**Authors:** Maria Ángeles Fernández de Dios, Olaia Iglesias, Marta Pazos, Maria Ángeles Sanromán

**Affiliations:** Chemical Engineering Department, University of Vigo, Isaac Newton Building, Campus As Lagoas, Marcosende, 36310 Vigo, Spain

## Abstract

The applicability of electro-Fenton technology to remediation of wastewater contaminated by several organic pollutants such as dyes and polycyclic aromatic hydrocarbons has been evaluated using iron-enriched zeolite as heterogeneous catalyst. The electro-Fenton technology is an advanced oxidation process that is efficient for the degradation of organic pollutants, but it suffers from the high operating costs due to the need for power investment. For this reason, in this study microbial fuel cells (MFCs) were designed in order to supply electricity to electro-Fenton processes and to achieve high treatment efficiency at low cost. Initially, the effect of key parameters on the MFC power generation was evaluated. Afterwards, the degradation of Reactive Black 5 dye and phenanthrene was evaluated in an electro-Fenton reactor, containing iron-enriched zeolite as catalyst, using the electricity supplied by the MFC. Near complete dye decolourization and 78% of phenanthrene degradation were reached after 90 min and 30 h, respectively. Furthermore, preliminary reusability tests of the developed catalyst showed high degradation levels for successive cycles. The results permit concluding that the integrated system is adequate to achieve high treatment efficiency with low electrical consumption.

## 1. Introduction

Green remediation reduces the demand placed on the environment during clean-up actions and avoids potential collateral environmental damage. Thus, the implementation of effective technologies for the remediation of hazardous organic pollutants in wastewaters plays a fundamental role. Therefore, this study focuses on the reduction of energy demand of the electro-Fenton degradation process by integration of alternative energy sources such as microbial fuel cells (MFCs).

In the last years, different advanced oxidation processes (AOPs) have proved to assess powerful oxidative techniques for several organic pollutants [1]. The AOPs depend on the *in situ* generation of hydroxyl radicals (^•^OH), a highly powerful oxidizing agent. These species are more effective oxidants (*E* ^0^ = +2.8 V) than the chemical reagents commonly adopted for this purpose, hypochlorous acid and permanganate (*E* ^0^≈ +1.5 V), and H_2_O_2_ (*E* ^0^ = +1.8 V).

Among the different AOPs, several researches have demonstrated that the electro-Fenton process is a promising technology to be more economical, efficient, and environmentally friendly to remove organic matter compared with conventional procedures [2–9]. In this process, the H_2_O_2_ is produced electrochemically via oxygen reduction on the cathode; then, the addition of ferrous ion into the system analogously generates the ^•^OH radicals in the classical Fenton's reaction. On the other hand, in this process, the ferrous ion is regenerated at the cathode, reducing its addition in comparison to the traditional Fenton's process [10].

Recently, the application of the electro-Fenton technology with iron heterogeneous catalysts has attracted the attention of different research groups [11–13]. The use of iron heterogeneous catalysts facilitates the reuse of iron; thereby several organic and inorganic matrixes have been used in order to obtain a stable iron catalyst to be used in heterogeneous Fenton reactions. In this field, our research group has recently made great progress and iron has been immobilized into alginate gel beads and sepiolite, showing in all cases high catalytic activity in the oxidation of several organic compounds with minimal iron leaching [11–13].

Therefore, the electro-Fenton process is efficient for the degradation of organic pollutants and it has been proved that this method is more effective than conventional Fenton's reagent; however, it suffers from the operational costs due to the need of power investment [2]. In order to reduce these costs, in a previous study [14], it was proved that the demand of energy could be supplied by sustainable alternative energy sources such as MFCs.

An MFC is a new type of fuel cells that offer the possibility of efficiently converting organic compounds into electricity with high energy conversion efficacy, mild operation conditions, and low cost substrates [15]. It has been around 100 years since the time of the first reported MFC [16] but only in recent years they have attracted substantial attention from research organizations; furthermore several breakthroughs have brought them closer to practical applications. Although the power generation from MFCs has improved considerably in recent years, it is still a big challenge [15, 17–19].

The performance of MFCs depends basically on intrinsic parameters such as system architecture, electrode material, bacterial community, and extrinsic parameters, for example, operating temperature, pH of the substrate, organic load, and so forth [20]. In the scale-up of an MFC extrinsic and intrinsic parameters play a significant role. For this reason, several attempts to increase the electricity generation from MFCs have been made, including modifications of the anode electrode material, alterations of the electrode design, biofilms, and the addition of mediators or a particulate substrate to the anode [21–24]. Therefore, these parameters will be evaluated in the present study in order to increase the yield and power densities that could, therefore, make it practical to use MFCs as power sources for an AOP such as the electro-Fenton process [14, 25].

Based on the facts mentioned above, the aim of this study is to enhance the power generation in MFCs by selection of an adequate anode and to use this energy to carry out the electro-Fenton treatment of organic pollutants using iron-enriched zeolite as catalyst. On the other hand, to determine the ability of the system to degrade different organic pollutants, the energy supplied by an MFC was applied to an external electro-Fenton process operating in batch mode.

## 2. Materials and Methods

### 2.1. MFC Design

**Figure 1:**
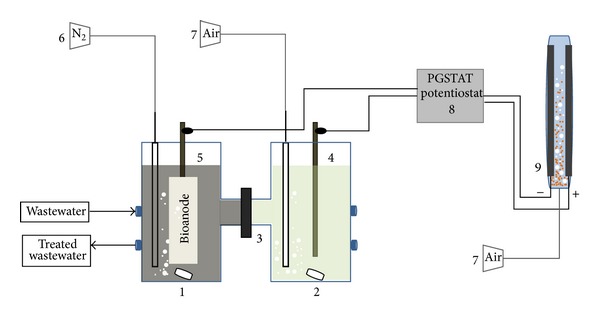
Experimental set-up; 1: anode chamber, 2: cathode chamber, 3: Sterion Cation Exchange Membrane, 4: cathode, 5: anode, 6: nitrogen supply, 7: air supply, 8: AutoLab PGSTAT 320 N potentiostat, and 9: electrochemical tubular glass reactor with graphite sheets and Fe-zeolite.

An H-type reactor MFC with two chambers of 250 mL separated by Sterion Cation Exchange Membrane was used (Figure 1). The electrodes were placed in each chamber in parallel with a gap between them of 12 cm. Graphite rod electrodes were used as cathode, and several anodes were tested: carbon cloth, graphite rod, rolled-up graphite sheet, and graphite rod combined with polyurethane foam or nylon fiber. The evolution of the power energy was recorded by AutoLab PGSTAT 302 N potentiostat.

### 2.2. MFC Operation

The anode chamber of the MFC was inoculated using the microorganisms present in the sewage sludge supplied by Telsar S.L. from an anaerobic urban wastewater treatment plant. After the initial proliferation, the anode chamber was continuously fed with synthetic wastewater with a defined medium composed of acetate (0.02 M) and a buffer solution (NH_4_Cl 0.31 g/L; KCl 0.31 g/L; NaH_2_PO_4_·2H_2_O 3.32 g/L; Na_2_HPO_4_·2H_2_O 10.32 g/L). The pH of the solution was initially adjusted to 7, and it was autoclaved at 121°C for 20 min. The cathode chamber was filled with tap water and the pH was adjusted to 2. Nitrogen and air were continuously pumped through anode and cathode compartments to maintain anaerobic or aerobic conditions, respectively. Both chambers maintained homogeneous conditions with magnetic stirrer bars.

### 2.3. Preparation of Iron-Enriched Zeolite (Fe-Zeolite)

The heterogeneous catalyst was prepared by iron adsorption onto natural zeolite (0.5–1 mm) provided by Tribar S.L.

For this purpose, several adsorption experiments were carried out at iron concentrations ranging between 100 and 1000 mg/L in order to investigate the kinetic behavior of the adsorption process. These experiments were carried out in 250 mL Erlenmeyer flasks by mixing a constant amount of zeolite (3 g) with a constant volume of iron aqueous solution (150 mL). The content in the flasks was agitated by placing them in a mechanical shaking incubator at 150 rpm and 20°C. Samples were taken periodically during the assays. Iron uptake was determined by the difference between the initial and final concentration in the aqueous solution. The uptake was calculated using the following equation:

(1) $$\begin{array}{c} q=\frac{\left(C_{0}-C\right)\cdot V}{W}, \end{array}$$

where *q* is the iron uptake (mg/g); *C*_0_ and *C* are the initial liquid-phase concentration of iron and the concentration through time in the solution (mg/L), respectively; *V* is the solution volume (L); *W* is the mass of adsorbent (g).

All the adsorption studies were repeated three times; the reported values are the average of those measurements.

### 2.4. Electro-Fenton Reactor Assisted by the MFC

The electro-Fenton reactor is a tubular glass reactor with a cylindrical body and a working volume of 15 mL (Figure 1). Experiments were carried out using anode and cathode graphite sheets with an immersed area of 10 cm^2^ and an electrode gap of 1.5 cm. The electrodes were fixed in caps and connected to the MFC in order to supply electricity to the electro-Fenton reactor. As it is mentioned above, the H_2_O_2_ is produced electrochemically by bubbling compressed air near the cathode at about 1 L/min. In addition, the air flow permits the fluidization of Fe-zeolite into the electro-Fenton reactor and the mixture of the reaction medium avoiding the concentration gradients in the reactor.

The reaction mixture contained Fe-zeolite at a final iron concentration of 150 mg/L in 15 mL of dye Reactive Black 5 solution (100 mg/L) or phenanthrene solution (18 mg/L). In these experiments, the pH was adjusted to 2 with sodium hydroxide or sulphuric acid and Na_2_SO_4_ (0.01 M) was used as electrolyte.

### 2.5. Analytical Procedures

#### 2.5.1. Sample Preparation

In all experiments, samples were taken periodically from the electro-Fenton reactor to be analyzed for pH and pollutant concentration. Samples were centrifuged at 10,000 rpm for 5 min, and the supernatant was separated from the zeolite to be analyzed. All the experiments and analytical determinations were done in duplicates, and the showed results are the mean values.

#### 2.5.2. Iron Determination in Liquid and Zeolite Samples

Iron in the liquid samples was determined with atomic absorption spectroscopy using the equipment Perkin Elmer SpectrAA-800.

The iron distribution in the Fe-zeolite was determined by scanning electron microscopy and energy dispersive spectrometry (SEM/EDS). This study was performed on a JEOL JSM-6700F equipped with an EDS Oxford Inca Energy 300 SEM using an accelerating voltage of 20 keV.

#### 2.5.3. Dye Removal Measure

The initial and residual dye concentrations were measured spectrophotometrically (V-630 UV-VIS-NIR, Jasco,) from 450 to 750 nm using a calibration curve associated with the area under the curve. Dye decolourization, expressed in terms of percentage, was calculated according to the following equation:

(2) $$\begin{array}{c} D=\frac{\left(A_{i}-A_{t}\right)100}{A_{i}}, \end{array}$$

where *D* is dye decolourization (%); *A*_*i*_ and *A*_*t*_ are area under the curve of the absorption spectrum from 450 nm to 750 nm at the initial time and through time, respectively.

#### 2.5.4. Phenanthrene Analysis

Phenanthrene concentration in the liquid samples was measured by reversed-phase high performance liquid chromatography (HPLC) equipped with a reversed-phase C8 column (150 × 4.6 mm, 5 *μ*m particle size, Zorbax Eclipse) with its corresponding guard column. The used HPLC system was an Agilent 1100 equipped with a quaternary pump and photodiode array UV/Vis detector (252.4 nm). 5 *μ*L of filtered sample (through a 0.45-*μ*m PVDF filter) was injected and then eluted from the column at a flow rate of 1 mL/min using an acetonitrile : water (60 : 40) as mobile phase.

#### 2.5.5. Anode Surface Characterization

A series of scanning electron microscopy (SEM) images were taken to provide a visual characterization of the consortium grown over the electrode. For microscopic observations, the anode was partially removed from each MFC. The samples were dehydrated; critical-point-dried, coated with gold, and then photographed. Images were collected on an FEI Helios Nanolab 600 DualBeam (FIB/SEM) (Electron Microscopy Service, C.A.C.T.I., University of Vigo).

### 2.6. Mathematical Fittings

In the different kinetics studies, the parameters were obtained by using the Sigma Plot 8.0 software that applies an iterative procedure, based on the Marquardt-Levenberg algorithm, which seeks the values of the parameters that minimize the sum of the squared differences between the observed and predicted values of the dependent variable.

## 3. Results and Discussion 

### 3.1. MFC Electricity Generation

Numerous microorganisms have the ability to transfer the electrons, derived from the metabolism of organic compounds, across their plasmatic membranes to the outside where they can be captured by an electrode which leads to the generation of a source of electrons. In addition, the selection of the inoculum source is a key parameter in the design of an MFC [26]. Kim et al. [27] reported that mixed cultures are more suitable for the development of MFCs due to the use of complex substrates such as wastewater. For this reason, in recent studies, the source of electroactive microorganisms has been domestic wastewater [28, 29] or anaerobic sewage sludge [17, 30, 31].

In this study microorganisms present in sewage sludge, supplied by Telsar S.L. from an anaerobic urban wastewater treatment plant, were used as electro-active microorganisms. As it is mentioned above, the anode material and its configuration play an important role in the power generation of MFCs. The electroactive biofilm can have significant impact on the yield and efficiency of the conversion processes. Thus, five kinds of anode: carbon cloth, graphite rod, rolled-up graphite sheet, and graphite rod combined with polyurethane foam or nylon fiber, were used in order to obtain an effective biofilm on the anode.

**Figure 2:**
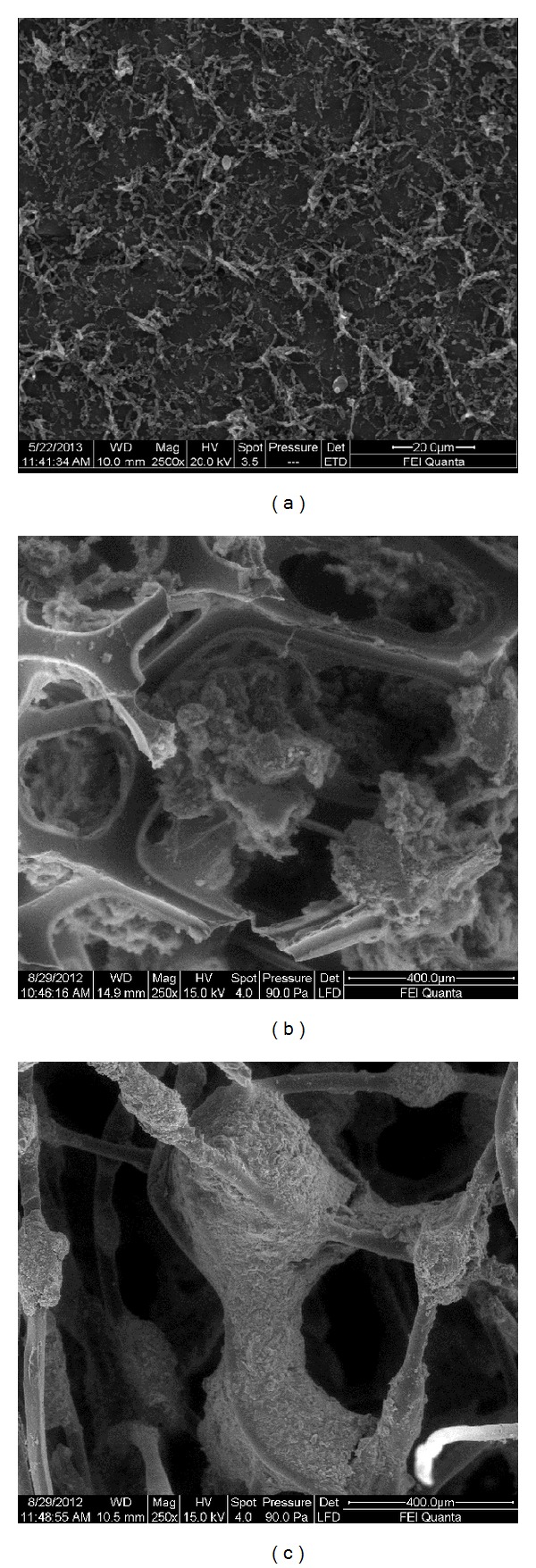
Scanning electron microscopy microphotographs of *electroactive microorganisms *grown on the anode. (a) Rolled-up graphite sheet, (b) polyurethane foam, and (c) nylon fiber.

All MFCs were left to run for several days at open circuit voltage (OCV) and the recorded voltage was increasing progressively until it reached a relatively stable potential which suggests that the anode was colonized by a stable bacterial community (Figure 2).

This fact is in agreement with the results reported by Fuentes-Albarrán et al. [32]. They found that the OCV increased progressively following a lag phase (presumably because bacteria are adapted to oxidize substrate) until it reached the same relatively stable potential. After that, the MFCs operated in closed circuit (CC) mode using a resistance. In Table 1, the different stable voltages reached in each configuration are shown. Values higher than 500 mV were obtained using rolled-up graphite sheet and graphite rod with polyurethane foam as electrode materials. In addition, the MFC using carbon cloth, as anode material, obtained the lowest voltage. These results indicated that the use of microorganisms present in sewage sludge from an anaerobic urban wastewater treatment plant could grow on rolled-up graphite sheet and generate stable electricity that could be used to supply electricity to drive an electro-Fenton process.

### 3.2. Iron Adsorption to Obtain Fe-Zeolite: Modelling of Kinetic Data and Characterization

Heavy metals such as iron, chromium, and manganese are toxic priority pollutants that commonly interfere with the beneficial use of wastewater for irrigation and industrial applications. Zeolites have high specific surface area, chemical and mechanical stability, layered structure, and high cation exchange capacity that makes these clays excellent low cost adsorbents [33, 34]. For this reason, in this study the sorption of iron from aqueous solutions onto natural zeolite has been studied with the purpose of obtaining an iron catalyst and also for removing iron from aqueous solutions.

The sorption kinetic is very important for the process design and the operational control of an adsorption process. In wastewater treatments, such kinetics studies are significant since they provide valuable insights into the reaction pathways and the mechanism of sorption reactions. This allows the description of the solute uptake which in turn controls the residence time of the sorbate in the solid-solution interface [35, 36].

**Figure 3:**
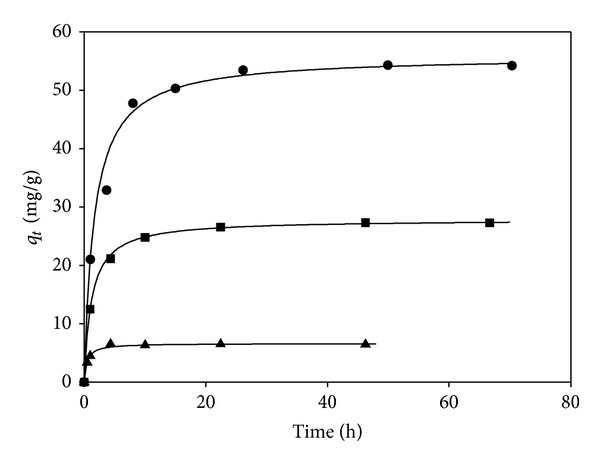
Adsorption kinetics of iron on zeolite at several values of iron concentration: filled circles 1000 mg/L, filled squares 500 mg/L, and filled triangles 100 mg/L. Lines indicate the simulated pseudo-second-order kinetic model.

In this study, the kinetic experiments were performed by varying the iron concentration between 100 and 1000 mg/L. The adsorption kinetics exhibited a rapid adsorption at low iron concentration and reached pseudo-adsorption-equilibrium after 20 h in all tested conditions (Figure 3). These results are in agreement with those obtained by Iglesias et al. [11] when iron was removed from simulated wastewater using sepiolite clay. The kinetics profile followed a behavior of pseudo-second-order which fits

(3) $$\begin{array}{c} q_{t}=q_{e}-\frac{q_{e}}{1+q_{e}\cdot k_{1}\cdot t}, \end{array}$$

where *q*_*t*_ (mg_Fe_/g_zeolite_) is the uptake at time *t* (h), *q*_*e*_ is the uptake at equilibrium, and *k*_1_ is the pseudo-second-order equilibrium rate constant (g_zeolite_/(mg_Fe_ · h)). The pseudo-second-order model provides an excellent fit between the predicted curves and the experimental values (*R* ^2^ > 0.99 in Figure 3).

These results are in accordance with previous reports [37], in which the mechanism of adsorption on clays is explained as a three-step mechanism: first, the adsorbate species migrate from the bulk liquid phase to the outer surface of adsorbent particles (film diffusion); secondly, the solvent species move within the micro and macropores of adsorbent particles (pore diffusion); and, in the end, the interaction between the adsorbate and the adsorbent species takes place within the surface.

**Figure 4:**
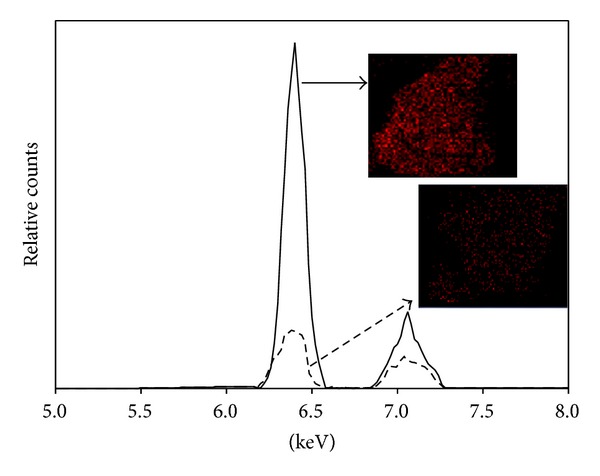
Energy dispersive spectral analysis and mapping (photos) of natural zeolite (medium dash line) compared with Fe-zeolite (solid line). The red points represent the iron distribution in the zeolites.

Therefore, these results indicate that the zeolite, a natural low cost adsorbent, can be successfully used for iron removal from aqueous solutions. However, more information about the iron distribution in the clay is necessary. Thus, to verify the iron adsorption and distribution onto zeolite, scanning electron microscopy and energy dispersive spectrometry (SEM/EDS) were performed. This equipment permits obtaining information about the surface of the sample topography and its chemical composition. In Figure 4, the energy peaks corresponding to the detected Fe by EDS spectral analysis and mapping of zeolite and Fe-zeolite are shown. In this figure, the Fe common peaks (*K*_*α*_ and *K*_*β*_) are readily resolved. These spectral analisys demostrate that the iron content increased after the adsorption process and its distibution is homogeneous onto zeolite. Therefore, by the use of zeolite as sorbent material, it is possible to obtain a double benefit: the removal of iron from aqueous solutions and the generation of Fe-zeolite that can be used as an iron heterogeneous catalyst.

### 3.3. Electro-Fenton Reactor Assisted by MFC

As it is reported by Brillas et al. [2] one of the main disadvantages of the application of AOPs, such as electrochemical or electro-Fenton technologies, is their high energy cost. In order to mitigate this problem, the application of the energy generated in the designed MFC for electro-Fenton processes was tested. When the nearly stable potential of the MFC was reached, different experimental tests were performed in order to determine the ability of this cell to permit the development of an electro-Fenton process to treat dye and polycyclic aromatic hydrocarbons (PAHs), Reactive Black 5 and phenanthrene, respectively.

**Figure 5:**
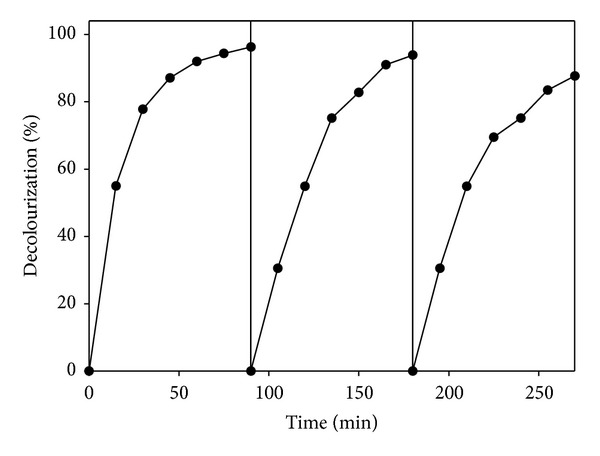
Decolourization profile of Reactive Black 5 using the electro-Fenton treatment in successive cycles.

Initially, the decolourization of Reactive Black 5 under the electro-Fenton process was carried out using Fe-zeolite as catalyst in the reactor described in Section 2. In order to determine the operational stability of Fe-zeolite for dye decolourization, successive cycles were performed. Dye decolourization along the time, after successive cycles using the same Fe-zeolite as catalyst, is shown in Figure 5. Near complete decolourization was reached after 90 min in the first cycle. As it can be observed in Figure 5, after 3 cycles, the time necessary to obtain the maximum dye decolourization was slightly increased, and the decolourization rate was not almost reduced in comparison to the first cycle. Finally, in order to evaluate the fixation of iron onto the zeolite, SEM and EDS analysis showed that the particles maintain their structure and keep the iron content. Thus, Fe-zeolite appeared to exhibit a high reusability with high stability. Furthermore, the homogenous distribution of iron on the particle of clay permits the electro-Fenton process to take place in successive cycles without the addition of Fenton agents.

After the good results achieved in the decolourization of Reactive Black 5, the same configuration was used to evaluate the degradation of phenanthrene. PAHs are an important kind of xenobiotic, persistent pollutants in several environments [38, 39]. Among them, phenanthrene has been widely used as an indicator and a model compound to study the degradation of this kind of pollutants [40, 41].

**Figure 6:**
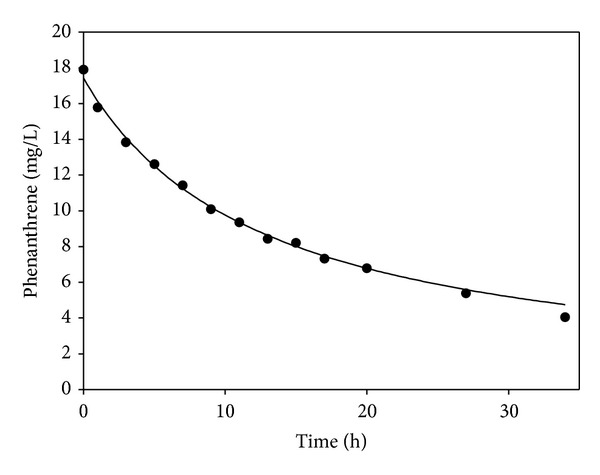
Electro-Fenton degradation of phenanthrene solution (18 mg/L). Lines indicate the simulated second-order kinetic model.

As shown in Figure 6, the second-order kinetic equation (4) fitted well the phenanthrene concentration profile along treatment time. Consider the following:

(4) $$\begin{array}{c} \frac{dC}{dt}=-k\cdot C^{2}, \end{array}$$

where *C* is concentration of phenanthrene (mg/L); *t* is time (h), and *k* is kinetic coefficient for the second-order reaction (L/mg·h).

The regression obtained for the model fits well (*R* ^2^  0.99) to Equation (4), and the kinetic coefficient was 0.0045 L/mg·h. These results confirm that the degradation rate is favoured when the phenanthrene concentration in the electro-Fenton reactor is high. Therefore, this technology could be adequate in the treatment of effluents with a high concentration of PAHs in a short time.

After the evaluation of both degradation processes, it is concluded that this combined technology, MFC-electro-Fenton process, may represent an environmentally friendly and cost competitive alternative.

## 4. Conclusions

This study demonstrates the potential application of the electro-Fenton process with Fe-zeolite to remediate polluted effluents when the power supply comes from a self-sustaining process such as MCFs. Based on the obtained results, it should be noted, that among the different kinds of tested anodes for improving the production of energy from the MFCs, rolled-up graphite sheet reached higher voltages. Furthermore, the obtained Fe-zeolite can be exploited as an iron heterogeneous catalyst for the electro-Fenton process without the external addition of Fenton's reagents. Therefore, the combination of MFCs, as an energy source, and electro-Fenton process with Fe-zeolite allows the degradation of organic pollutants in a more efficient and environmentally friendly way.

## Figures and Tables

**Table 1:** Maximum voltage values reached.

Anode	Maximum voltage (mV)
Carbon cloth	216.6
Graphite rod	310.4
Rolled-up graphite sheet	614.3
Graphite rod + polyurethane foam	523.3
Graphite rod + nylon fiber	410.1
